# Stability and compatibility of phentolamine mesylate combined with furosemide in common infusion solutions for pediatric critical care

**DOI:** 10.3389/fped.2026.1893184

**Published:** 2026-07-29

**Authors:** Baoxia Fang, Ruiling Shang, Fuchao Chen, Yanhong Liu, Huimin Liu

**Affiliations:** 1 Sinopharm Dongfeng General Hospital, Hubei University of Medicine, Shiyan, China; 2 Renmin Hospital, Hubei University of Medicine, Shiyan, Hubei, China

**Keywords:** furosemide, pediatric intensive care units, phentolamine, stability, syringe

## Abstract

**Background:**

Intravenous phentolamine mesylate and furosemide are widely administered via infusion pumps to treat severe pediatric critical illnesses. Their off-label concurrent use lacks reliable physicochemical compatibility and stability data, bringing potential medication safety risks. This study aimed to evaluate the 8-hour compatibility and stability of the two drugs mixed in syringes under simulated clinical *in vitro* conditions.

**Methods:**

Two clinically relevant concentration gradients were set for testing: phentolamine mesylate at 0.1 and 0.5 mg·mL⁻^1^, and furosemide at 0.5 and 1.0 mg·mL⁻^1^. Drug admixtures were prepared by dilute mixing using either 0.9% sodium chloride injection (0.9% NS) or 5% glucose injection (5% GS). All solutions were stored at room temperature (25 ± 1 °C) under natural light and sampled at 0, 2, 4, 6 and 8 h. Physicochemical indicators including appearance, pH value, insoluble particles and relative drug content were determined to assess mixture stability.

**Results:**

Throughout the 8-hour observation period, all mixed solutions remained clear and colorless without precipitation, turbidity, or discoloration. The pH values remained stable with a fluctuation range within ±0.4 units. The number of insoluble particles complied with the standards specified in the 2020 edition of the Chinese Pharmacopoeia. Moreover, the relative percentage content of both phentolamine mesylate and furosemide remained above 95% at all time points.

**Conclusion:**

Under *in vitro* conditions at room temperature (25 ± 1 °C) with natural light exposure, admixtures of phentolamine mesylate (0.1 or 0.5 mg·mL⁻^1^) and furosemide injection (0.5 or 1.0 mg·mL⁻^1^) prepared via dilute mixing in either 0.9% NS or 5% GS remained physicochemically stable for up to 8 h. The findings are only valid for the specific experimental parameters in this work and cannot be generalized to routine off-label combined administration in pediatric critical care.

## 1 Introduction

Childhood pneumonia ranks among the most prevalent respiratory disorders in pediatric populations worldwide. The World Health Organization reported that approximately 150 million children under 5 years old develop pneumonia annually, and severe pneumonia remains the leading cause of hospitalization and mortality for infants younger than 2 years globally (1). Immature organ function across multiple systems in young children renders them highly vulnerable to multi-organ complications following severe pulmonary infection, among which heart failure poses severe threats to pediatric survival and long-term health (2).

Standard therapeutic regimens for children with pneumonia combined with heart failure consist of anti-infective therapy, diuretics, cardiotonic agents, oxygen supplementation and bronchodilator interventions (3, 4). Published cohort studies confirm that co-administration of intravenous phentolamine mesylate and furosemide via continuous infusion pump for the treatment of childhood pneumonia complicated with heart failure effectively reduces peripheral vascular resistance, improves cardiac performance and pulmonary gas exchange, and relieves pulmonary edema and respiratory failure (5–7). This vasodilator-diuretic admixture is also widely adopted for managing other high-risk pediatric critical conditions, including refractory heart failure, cor pulmonale and neonatal respiratory distress syndrome (8, 9).

Notably, pediatric intensive care units (PICUs) face unique clinical administration barriers that force nurses to compound multiple injectable medications within single syringes or shared venous lumens. Retrospective observational data demonstrated that PICU patients receive an average of 6.9 concurrent infusions with merely 3.8 available catheter lumens per patient day, forcing frontline nurses to compound multiple injectable drugs in the same syringe for simultaneous pump delivery (10). Separate venous lines for each agent are frequently unavailable, compelling bedside nurses to prepare admixtures of vasoactive and diuretic drugs in advance within disposable syringes for prolonged pump delivery. This routine off-label compounding workflow creates unavoidable physicochemical interaction risks, as frontline nurses lack standardized, evidence-based mixing protocols to avoid visible precipitates or subvisible micro-particles generated during preparation and prolonged bedside storage (11).

Furosemide is well-documented as a high-risk agent for physical incompatibility in critical care settings. Direct mixing of furosemide with many commonly used intravenous agents, including antibiotics, antiemetics and vasoactive medications, rapidly triggers instantaneous turbidity, opalescence or white particulate precipitation (12, 13). These precipitates and invisible micro-particles may block thin pediatric central venous catheters, trigger pulmonary microembolism, lower effective drug concentration and weaken therapeutic efficacy, potentially inducing severe vascular adverse events in fragile critically ill children (14). Importantly, existing compatibility documents for furosemide merely record observations of instantaneous Y-site mixing, lacking quantitative stability data under pediatric clinical concentrations and 8-hour prolonged storage conditions.

Despite proven pharmacological synergy, the phentolamine-furosemide admixture represents a typical off-label combined regimen without commercially available premixed infusion products. Clinical compounding must be performed either in centralized intravenous infusion centers or directly at ward nursing stations prior to pump infusion. Systematic retrieval of drug package inserts, Trissel's Injectable Drug Compatibility Handbook, and mainstream biomedical databases reveals severe scarcity of targeted stability data for this specific dual-drug combination (9, 15). Therefore, this study prepared diluted phentolamine mesylate and furosemide admixtures with 0.9% sodium chloride (0.9% NS) or 5% glucose injection (5% GS). Under simulated clinical conditions, the physicochemical stability of these admixtures was assessed over an 8-hour period through evaluation of solution appearance, pH value, insoluble particles and drug residual content, aiming to provide practical evidence supporting the safe off-label application of these combinations in pediatric critical care settings.

## 2 Materials and methods

### 2.1 Drugs and reagents

Phentolamine mesylate (purity 99.7%, lot# 100110-201103) and furosemide (purity 99.8%, lot# 100544-202104) reference substance were purchased from the National Institute for the Control of Pharmaceutical and Biological Products (Beijing, China). Phentolamine mesylate injection (1 mL: 10 mg, lot# D230603, Shanghai Xudong Haipu Pharmaceutical Co., Ltd.), Furosemide injection (2 mL: 20 mg, lot# 047230713, Guangzhou Real Pharmaceutical Technology Co., Ltd.), 0.9% NS (250 mL: 2.25 g, lot# 23052747, Dalian Otsuka Pharmaceutical Co., Ltd.), and 5% GS (250 mL: 12.5 g, lot# 23101941, Dalian Otsuka Pharmaceutical Co., Ltd.) were purchased from Sinopharm Holding Company Limited (Hubei, China). HPLC grade acetonitrile were purchased from Tianjin Kemiou Chemical Reagent Co., LTD. (Tianjing, China). Potassium dihydrogen phosphate (KH_2_PO_4_) were purchased from Tianjin Bodi Technology Development Co., Ltd. (Tianjin, China).

### 2.2 Instruments and chromatographic conditions

The HPLC system (Agilent 1260) consisted of Infinity VL type four element low pressure gradient pump, Infinity standard automatic sampler, Infinity column temperature chamber, Infinity VL Plus variable wavelength detector (G1314C) and OpenLab CDS 2.8 software. The chromatographic column was Shim-pack GIST C_18_ column (4.6 mm × 150 mm, 5 μm). A mobile phase consisted of acetonitrile—0.05 mol·L⁻^1^ KH_2_PO_4_ (30/70, V/V) at a flow rate of 1.0 mL·min⁻^1^. The column was thermostated at 30 °C and injection volume was 20 μL during analysis. The detection wavelength were 280 nm for phentolamine mesylate and furosemide.

### 2.3 Preparation of solutions

#### 2.3.1 Preparation of standard solution

Accurately weigh appropriate quantities of reference standards for phentolamine mesylate and furosemide separately, and transfer them into individual 25 mL volumetric flasks. Dilute with the mobile phase to the calibration mark to prepare stock solutions of the reference standards, each with a concentration of 1.0 mg·mL⁻^1^. Store these stock solutions in a freezer at −20 °C for subsequent use. Subsequently, accurately pipette 2 mL of each stock solution and combine them into a single 10 mL volumetric flask. Adjust the volume to the calibration mark with the mobile phase to prepare a mixed standard solution containing both substances at a concentration of 200 μg·mL⁻^1^.

#### 2.3.2 Preparation of the mixture of phentolamine mesylate and furosemide

According to drug package inserts and standard administration protocols for PICU (3, 4, 8, 9), all test admixtures were prepared via a dilute mixing technique. The step-by-step procedure for the combination solution is as follows.

1. A 50 mL sterile disposable syringe was used to withdraw 40 mL of 0.9% NS or 5% GS to prepare a buffered base diluent.
2. The required volume of phentolamine mesylate injection (per concentration gradients in Table 1) was slowly added to the prefilled diluent syringe, followed by repeated gentle inversion to ensure full homogenization.
3. With the phentolamine mesylate fully diluted, extract the corresponding volume of furosemide injection, add it into the syringe, and invert gently and repeatedly for uniform mixing.
4. Top up the mixture with matching blank diluent (0.9% NS or 5% GS) to a total volume of 50 mL, then invert thoroughly one final time to complete constant volume. Visually inspect the final solution for clarity, turbidity, precipitation or discoloration before clinical infusion.

**Table 1:** Drug formulation of mixed solutions containing phentolamine mesylate and furosemide.

Mixture no.	Phentolamine mesylate	Furosemide	Total volume
0.9% Sodium chloride injection (0.9% NS)			
1	5 mg (0.5 mL)	25 mg (2.5 mL)	50 mL
2	5 mg (0.5 mL)	50 mg (5.0 mL)	50 mL
3	25 mg (2.5 mL)	25 mg (2.5 mL)	50 mL
4	25 mg (2.5 mL)	50 mg (5.0 mL)	50 mL
5% Glucose injection (5% GS)			
1	5 mg (0.5 mL)	25 mg (2.5 mL)	50 mL
2	5 mg (0.5 mL)	50 mg (5.0 mL)	50 mL
3	25 mg (2.5 mL)	25 mg (2.5 mL)	50 mL
4	25 mg (2.5 mL)	50 mg (5.0 mL)	50 mL

Three independent replicate admixtures (*n* = 3) were prepared for each concentration group following the four-step compounding procedure described above. After constant volume, the plunger of each disposable syringe was fully pushed to expel residual air, and the syringe tip was then securely sealed with a sterile rubber cap to prevent both solvent evaporation and external contamination. All capped syringes were stored under natural light at a controlled ambient temperature of (25 ± 1) °C, followed by an 8-hour continuous physicochemical stability assessment.

### 2.4 Validation of HPLC analytical method

#### 2.4.1 System suitability test

A 20 μL mixed standard solution of phentolamine mesylate and furosemide, sample solution and a blank solution were separately injected into the HPLC system in accordance with the chromatographic conditions described in Section 2.2. Mobile phase composition and column temperature were optimized to evaluate the resolution, theoretical plate number and retention behavior of phentolamine mesylate and furosemide in mixed standard solutions.

#### 2.4.2 Linearity

In the linear range, take the mixed standard solution of phentolamine mesylate and furosemide and put it at room temperature. After mixing it well with ultrasound, accurately absorb an appropriate solution with a pipette into different 10 mL bottles and dilute it with the mobile phase to set the volume. A series of standard solutions containing phentolamine mesylate with a concentration of 0.05–0.20 mg·mL^−1^ and furosemide with a concentration of 0.01–0.20 mg·mL^−1^ were prepared. Each standard solution (20 μL) was injected under the aforementioned chromatographic conditions, and corresponding peak areas were recorded. Linear regression analysis was performed with analyte concentration (*X*) as the abscissa and peak area (*Y*) as the ordinate to acquire linear regression equations and correlation coefficients (*r*).

#### 2.4.3 Precision

Quality control (QC) standard solutions of phentolamine mesylate (50.0, 100.0 and 150.0 μg·mL^−1^, respectively) and furosemide (50.0, 100.0 and 150.0 μg·mL^−1^, respectively) under Section 2.4.2 was taken for 6 consecutive samples on the same day for determination. The peak area RSD was calculated to investigate the intra-day precision. The inter-day precision of the instrument was examined by sampling the same concentration of QC solution for 3 consecutive days.

#### 2.4.4 Stability

For the stability test, two kinds of drug sample solutions were prepared. They were injected and analyzed at 0, 1, 2, 4, 6, and 8 h, respectively. Peak areas of each chromatogram were recorded, and the drug contents and RSD value were calculated to investigate the stability of the sample solution.

#### 2.4.5 Recovery

For recovery testing, the standard solutions were added to the mixture of phentolamine mesylate and furosemide with a known content (high, medium and low concentration levels of 50%, 100% and 150%, respectively). After thorough mixing and volumetric dilution, each solution was injected for analysis. The rate of each drug recovery rate and RSD value were calculated by subtracting the known amount from the measured amount and then dividing by the added amount.

### 2.5 Compatibility and stability investigation

#### 2.5.1 Appearance

At 0, 2, 4, 6, and 8 h, 5 mL of each mixture solution was transferred into a clean and transparent 10 mL Nessler color comparison tube. An equal volume of 0.9% NS or 5.0% GS was set as the blank control. All solutions were visually inspected for discoloration, bubbles, turbidity and precipitates.

#### 2.5.2 pH value and insoluble particles

The pH value of admixtures was measured with a PHS-3C pH meter (INESA Scientific Instrument Co., Ltd., Shanghai, China). At each scheduled time points, 5 mL solution was sampled, for insoluble particle counting via the microscopic method. Each solution was measured in triplicate, and the results are reported as the mean ± standard deviation (SD).

#### 2.5.3 Determination of drug content

Aliquots of each mixture were diluted 5-fold with the corresponding solvent and filtered through a 0.45 μm microporous membrane. A volume of 20 μL filtrate was injected and analyzed by the above chromatographic conditions. Each sample was repeated three times, and the drug content at different time points is calculated. The drug content at 0 h is 100%. The relative percentage content changes of different drugs at each time point were calculated. Results were expressed as mean ± SD.

#### 2.5.4 Statistical description and stability judgment criteria

All stability assessments in this study adopted predefined descriptive acceptance criteria without inferential statistical tests. Admixtures were deemed physically stable at all sampling time points over 8-hour storage if no visible turbidity or precipitates were observed, particulate matter met pharmacopoeial limits (≤12 particles ≥10 μm and ≤2 particles ≥25 μm per 1 mL), and pH varied within a 10% relative range from baseline. Chemical stability was validated when both analytes retained no less than 90% of their initial concentrations at every detection time point throughout storage.

## 3 Results

### 3.1 Results of HPLC analytical method validation

**Figure 1:**
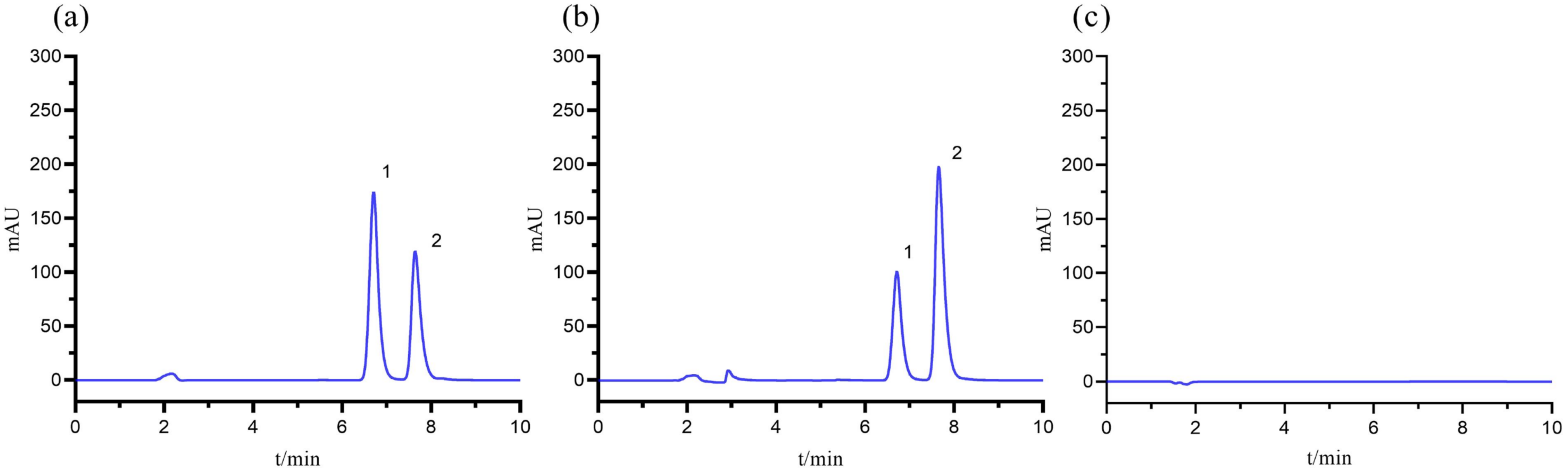
HPLC chromatogram of phentolamine mesylate (1) and furosemide (2). **(a)** Mixed reference solution; **(b)** sample of phentolamine mesylate and furosemide mixture; **(c)** blank control.

Typical HPLC chromatograms of mixed standard solution, blank solution and test sample solution are displayed in Figure 1. The retention times of phentolamine mesylate and furosemide were 6.7 and 7.6 min, respectively. No interfering peaks were observed at the retention positions of the two analytes in the blank diluent chromatogram, demonstrating the absence of matrix interference. Satisfactory resolution between phentolamine mesylate and furosemide was achieved, which fulfilled the quantitative detection requirements for the two target compounds. The linear regression equations for phentolamine mesylate and furosemide were *Y* = 33.639X-2.691 (*R*^2^ = 0.9999) and *Y* = 90.253X- 35.602 (*R*^2^ = 0.9998) with linear concentration ranges of 5.0–200.0 and 10.0–200.0 μg·mL⁻^1^ correspondingly. The RSD of intra-day and inter-day peak area was less than 2.0%, confirming the good precision of the established assay. The RSD values for the peak areas of phentolamine mesylate and furosemide were 1.1% and 1.2%, suggesting that the sample solutions maintained stability within an 8-hour period. The mean recovery values of phentolamine mesylate and furosemide were 100.2% and 99.8%, with corresponding RSDs of 0.9% and 1.3%, which verified reliable accuracy of the method for simultaneous quantification of the two analytes.

### 3.2 The results of the compatibility and stability

**Figure 2:**
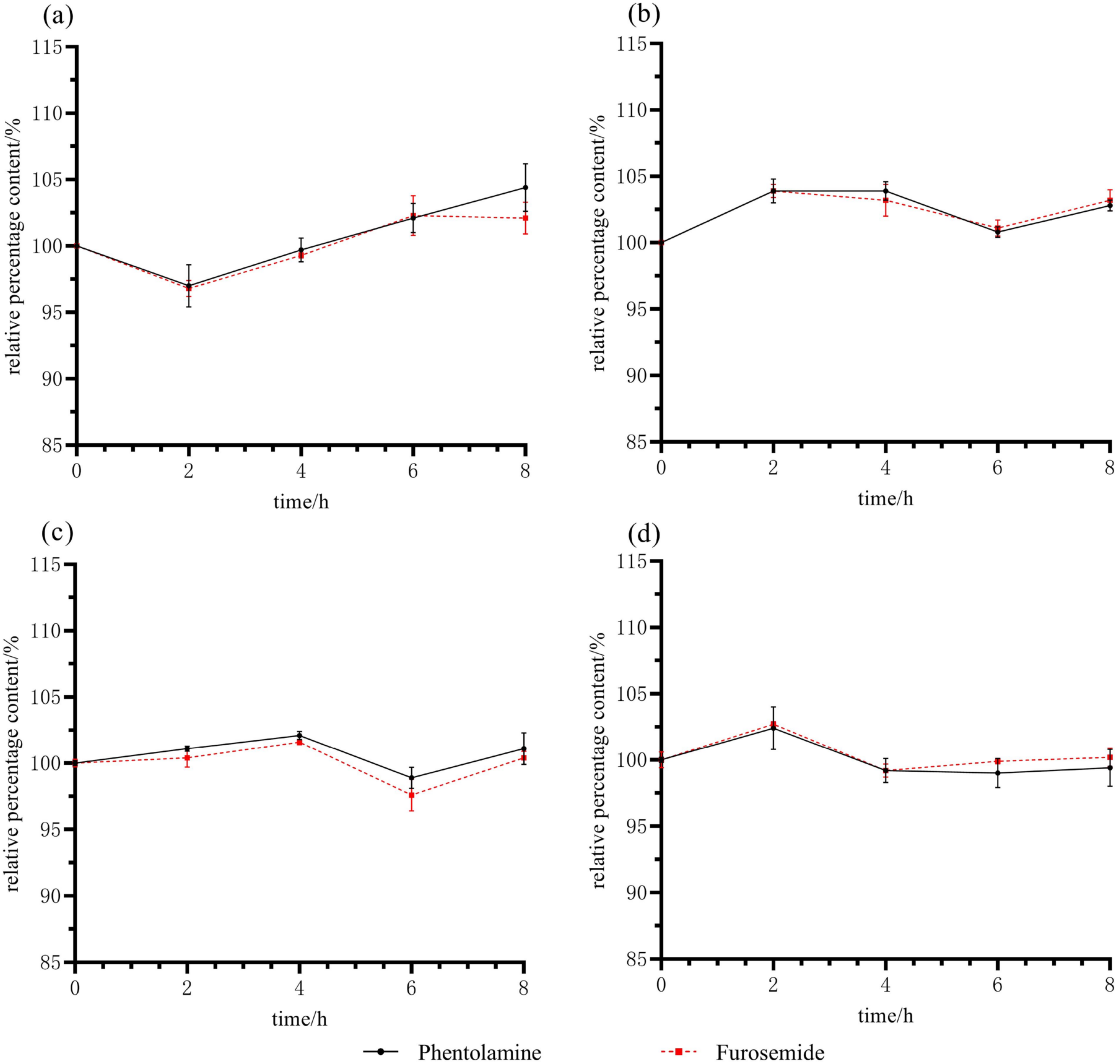
Relative percentage of phentolamine mesylate and furosemide in 0.9% NS during 8 h (mean ± SD, *n* = 3 independent preparation replicates). **(a)** 0.1 mg/mL phentolamine-0.5 mg/mL furosemide; **(b)** 0.1 mg/mL phentolamine-1.0 mg/mL furosemide; **(c)** 0.5 mg/mL phentolamine-0.5 mg/mL furosemide; **(d)** 0.5 mg/mL phentolamine-1.0 mg/mL furosemide.

**Figure 3:**
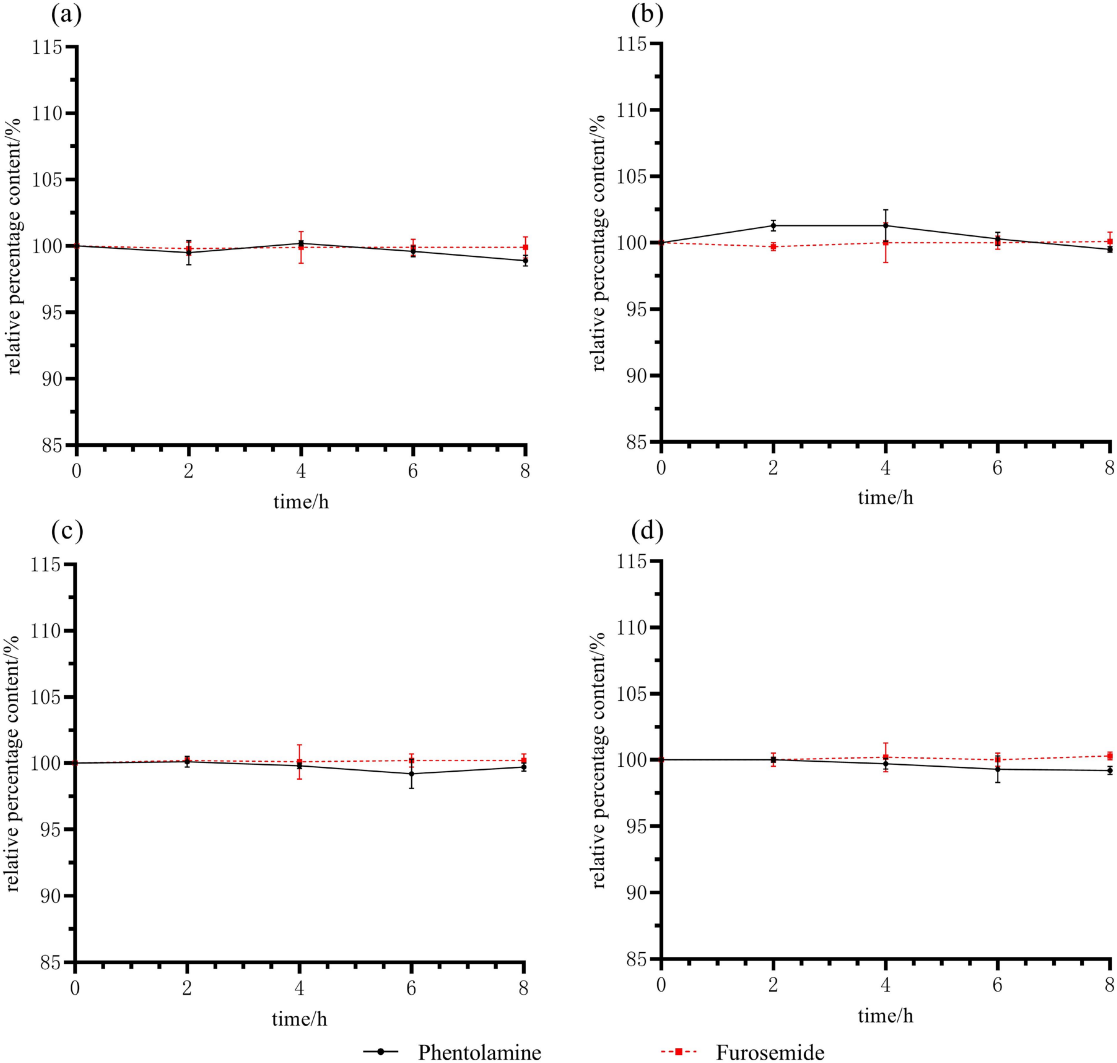
Relative percentage of phentolamine mesylate and furosemide in 5% GS during 8 h (mean ± SD, *n* = 3 independent preparation replicates). **(a)** 0.1 mg/mL phentolamine-0.5 mg/mL furosemide; **(b)** 0.1 mg/mL phentolamine-1.0 mg/mL furosemide; **(c)** 0.5 mg/mL phentolamine-0.5 mg/mL furosemide; **(d)** 0.5 mg/mL phentolamine-1.0 mg/mL furosemide.

During the 8-hour experiment, all mixtures remained colorless, clear and transparent, with no precipitation or color change observed. The pH value fluctuated within ±0.4 pH units (Table 2), demonstrating that the pH of the diuretic mixture composed of four different formulations remained stable over the 8-hour period. As shown in Table 3, the quantity of insoluble particles ≥10 and ≥25 μm in all admixture groups at every sampling time point (0, 2, 4, 6, 8 h) met the standards specified in the 2020 edition of the Chinese Pharmacopoeia, i.e., less than 12 particles ≥10 μm and less than 2 particles ≥25 μm per 1 mL solution. The content changes of phentolamine mesylate and furosemide injection in 0.9% NS or 5% GS at various time points are shown in Figures 2, 3, respectively. As shown in Figures 2, 3, the relative contents of the two drugs in different mixtures were ≥95%, indicating that the mixtures can remain stable within 8 h under the light condition and room temperature (25 ± 1) °C.

**Table 2:** pH value change of phentolamine mesylate and furosemide mixtures within 8 h (mean ± SD, *n* = 3 independent preparation replicates).

Serial number of the mixture	pH value				
	0 h	2 h	4 h	6 h	8 h
0.9% Sodium chloride injection (0.9% NS)					
1	7.63 ± 0.09	7.51 ± 0.07	7.46 ± 0.16	7.44 ± 0.06	7.43 ± 0.13
2	8.15 ± 0.11	8.08 ± 0.05	8.00 ± 0.04	7.96 ± 0.22	7.91 ± 0.15
3	7.06 ± 0.24	7.10 ± 0.04	7.13 ± 0.12	7.18 ± 0.14	7.21 ± 0.06
4	7.27 ± 0.13	7.42 ± 0.17	7.54 ± 0.21	7.51 ± 0.15	7.65 ± 0.18
5% Glucose injection (5% GS)					
1	7.28 ± 0.12	7.56 ± 0.09	7.57 ± 0.14	7.55 ± 0.23	7.61 ± 0.10
2	8.12 ± 0.13	8.11 ± 0.04	8.07 ± 0.17	8.06 ± 0.08	8.01 ± 0.25
3	6.88 ± 0.06	6.92 ± 0.24	6.90 ± 0.06	6.96 ± 0.13	7.04 ± 0.06
4	7.34 ± 0.08	7.35 ± 0.11	7.39 ± 0.12	7.42 ± 0.05	7.52 ± 0.11

**Table 3:** Insoluble particles of phentolamine mesylate and furosemide mixtures within 8 h (mean ± SD, *n* = 3 independent preparation replicates).

Time (h)	Mixture 1		Mixture 2		Mixture 3		Mixture 4	
	≥10 μm	≥25 μm	≥10 μm	≥25 μm	≥10 μm	≥25 μm	≥10 μm	≥25 μm
0.9% Sodium chloride injection (0.9% NS)								
0	6.3 ± 2.3	0.3 ± 0.6	8.7 ± 2.1	0.0 ± 0.0	5.0 ± 3.0	0.7 ± 0.6	7.7 ± 2.5	0.7 ± 0.6
2	4.0 ± 1.7	0.0 ± 0.0	3.7 ± 1.5	1.0 ± 1.0	8.3 ± 1.5	0.3 ± 0.6	7.0 ± 2.0	1.0 ± 1.0
4	7.3 ± 2.5	0.3 ± 0.6	6.3 ± 1.5	0.7 ± 0.6	8.0 ± 1.7	0.0 ± 0.0	6.3 ± 1.5	0.3 ± 0.6
6	4.7 ± 0.6	1.3 ± 0.6	4.0 ± 1.0	0.3 ± 0.6	6.3 ± 1.2	1.0 ± 1.0	5.0 ± 1.7	0.7 ± 1.2
8	3.3 ± 0.6	0.7 ± 0.6	4.3 ± 2.1	0.0 ± 0.0	3.7 ± 0.6	0.3 ± 0.6	8.0 ± 1.0	0.7 ± 0.6
5% Glucose injection (5% GS)								
0	3.7 ± 2.1	0.0 ± 0.0	4.7 ± 0.6	1.0 ± 1.0	3.3 ± 1.2	0.7 ± 1.2	6.0 ± 1.7	0.0 ± 0.0
2	3.0 ± 1.7	0.0 ± 0.0	2.7 ± 0.6	1.0 ± 0.0	5.0 ± 2.6	0.3 ± 0.6	7.7 ± 0.6	1.0 ± 1.0
4	7.3 ± 3.1	0.3 ± 0.6	3.3 ± 2.1	0.0 ± 0.0	6.7 ± 2.1	0.0 ± 0.0	2.3 ± 1.5	0.0 ± 0.0
6	3.7 ± 1.5	0.0 ± 0.0	5.0 ± 2.0	0.3 ± 0.6	3.3 ± 0.6	0.0 ± 0.0	4.7 ± 1.5	1.0 ± 1.0
8	5.3 ± 1.5	0.3 ± 0.6	5.3 ± 1.5	0.3 ± 0.6	2.3 ± 0.6	0.3 ± 0.6	2.0 ± 0.0	0.0 ± 0.0

## 4 Discussion

### 4.1 Rational evaluation of the combination of phentolamine mesylate and furosemide

Phentolamine mesylate is a relatively short—acting α-adrenergic receptor blocker. It can dilate blood vessels, reduce peripheral vascular resistance, enhance myocardial contractility, increase blood volume, and improve microcirculation. Clinically, it is mainly used to treat heart failure with pulmonary congestion or pulmonary edema, vasospastic diseases, septic shock, and other conditions (7, 8). Furosemide is a high-efficacy loop diuretic, suitable for the treatment of edema associated with congestive heart failure, cirrhosis, and kidney diseases (including nephrotic syndrome) in adult and pediatric patients. According to literature reports, the combination of phentolamine mesylate and furosemide is widely used in the treatment of refractory heart failure in children or adults, acute renal failure, chronic cor pulmonale with heart failure, bronchopneumonia, severe pneumonia, and pneumonia with heart failure (3, 4, 9). The combined use of the two drugs has a synergistic effect in improving cardiac and pulmonary respiratory functions and reducing edema symptoms. When combining drugs, drug—drug interactions need to be considered. By searching the DrugBank Online database (https://go.drugbank.com), it is suggested that when the two drugs are used together, phentolamine may reduce the antihypertensive activity of furosemide. Therefore, it is recommended to monitor the patient's blood pressure frequently during clinical application. Based on the above analysis, the compatibility prescription of phentolamine mesylate and furosemide injections is rational.

### 4.2 Analysis on the stability of the mixture of phentolamine mesylate and furosemide

In this study, simulating the clinical medication regimen, the stability of the mixture of phentolamine mesylate and furosemide injections in a disposable syringe was investigated within 8 h under the light condition at room temperature. The results showed that the mixture solution remained colorless, clear, and transparent during the study period, with no precipitation or color change. The pH of the mixture solution was between 6.8 and 8.2, showing no obvious change. The insoluble particles met the requirements of the pharmacopoeia (Table 3), and the relative percentages of the two drugs were relatively stable. However, in the preliminary experiments of this study, when phentolamine mesylate injection and furosemide injection were mixed, regardless of the order in which they were combined, white precipitate formation was observed upon contact between the two injections. This precipitation phenomenon became more pronounced as the concentration ratio of furosemide to phentolamine mesylate injection increased, indicating potential incompatibility between these drug injections. However, when 0.9% NS or 5% GS was added to the turbid or precipitated mixture, the amount of precipitate gradually decreased until it disappeared with increasing solvent volume. The immediate white precipitate produced following direct mixing of undiluted phentolamine mesylate and furosemide injections may be attributable to pH-dependent shifts in the ionization equilibrium of the two active pharmaceutical ingredients, a phenomenon that is likely related to their respective pKa values. Phentolamine mesylate bears a protonatable imidazoline moiety with a pKₐ of 7.7; its commercial injection is formulated at pH 2.5–5.0, a range substantially lower than its pKₐ, so the drug predominantly exists as water-soluble, positively charged cations. In contrast, furosemide exhibits a pKₐ of 3.9, and its marketed injection is alkalinized with sodium hydroxide to pH 8.5–9.0, stabilizing furosemide in its deprotonated, soluble anionic state (16). When the two concentrated stock solutions are combined directly, the strongly alkaline furosemide formulation rapidly raises the bulk pH above 7.7-the critical pKa threshold of phentolamine mesylate. This abrupt pH shift may drive rapid deprotonation of cationic phentolamine mesylate, which would convert the water-soluble ionic species into neutral lipophilic free base with markedly low aqueous solubility; such a solubility change could potentially account for the visible precipitate formation. However, dilution with an infusion solvent causes relatively minor changes in the pH of the resulting mixture, thereby reducing or preventing precipitation. Therefore, in this study, a diluted preparation method was adopted for the formulation of the mixture solutions: first, a sufficient amount of 0.9% NS or 5% GS was added, followed by one drug injection after thorough mixing, and then the second drug injection was added.

Clinically, the combination of two or more drugs is frequently employed to achieve synergistic effects or reduce adverse reactions. However, when multiple drugs are mixed, physical and chemical reactions may occur in the resulting solution due to differences in the physicochemical properties of the drugs, potentially causing drug degradation and loss of efficacy. Some literature (15, 16) has studied the compatibility and stability of phentolamine mesylate with other drugs and found that phentolamine can remain stable in 0.9% NS or 5% GS when combined with pituitrin, aminophylline, and diprophylline injection. Phentolamine mesylate contains a phenolic hydroxyl group in its molecular structure, which is susceptible to oxidation and discoloration under alkaline conditions, with color intensity increasing over time. Furosemide injection, a potent diuretic widely used in clinical practice, exhibits numerous incompatibilities. For example, when used in combination with amiodarone, ambroxol, aminomethylbenzoic acid, azithromycine, blinatumomab (17), caspofungin acetate, ceftriaxone, chlorpromazine, cisatracurium besylate, cimetidine, diazepam, dobutamine, droperidol, esmolol, haloperidol lactate, heparin (18), erythromycin lactobionate, famotidine, gentamicin sulfate, ketamine, lansoprazole, metoclopramide, mehydroxyamine, midazolam (19), omeprazole, ondansetron, promethazine, quinolone antibiotics, telavancin hydrochloride, thiopental sodium, urapidil, vasopressin, vancomycin, etc., white turbidity, milky suspension, flocculent precipitates, or liquid discoloration may occur (17–30).

These phenomena can primarily be attributed to two factors. First, furosemide injection is inherently alkaline, and mixing it with acidic drugs may lead to changes in pH or ionic strength, reducing drug solubility and causing precipitation. Second, furosemide may chemically react with other drugs to form insoluble substances or alter their physicochemical properties. Nevertheless, a clear divergence in compatibility behavior is observed between the diluted stable admixtures of furosemide and phentolamine mesylate characterized in this study and previously documented cases of furosemide incompatibility. This distinction emerges from a systematic evaluation of four critical physicochemical parameters, such as pH environment, dilution ratio, final drug concentration, and mixing sequence. Earlier compatibility studies involving furosemide and acidic intravenous agents predominantly employed Y-site administration simulations, wherein equal volumes (1:1) of high- concentration drug infusions were combined. This approach frequently precipitated abrupt pH shifts and immediate formation of insoluble furosemide free-base precipitates. In contrast, the current study utilized large-volume 0.9% NS or 5% GS as buffering diluents, which functioned as effective pH buffers. The abundant diluent effectively mitigated drastic pH fluctuations and fully dissolved poorly soluble neutral drug molecules, which also accounted for the complete disappearance of precipitates observed in preliminary trials after supplementary addition of 0.9% NS or 5% GS to turbid undiluted admixtures. Under undiluted mixing conditions, the addition sequence of phentolamine mesylate and furosemide exerted no protective effect, and white precipitates emerged immediately upon contact of the two stock solutions regardless of administration order, which was consistent with published incompatibility findings of furosemide paired with midazolam and amiodarone (19). Notably, mixing furosemide with aminomethylbenzoic acid or dobutamine triggers irreversible chemical reactions including drug decomposition and polymerization, generating novel insoluble derivatives (9, 22). This type of chemically mediated incompatibility cannot be eliminated merely by dilution, highlighting the unique clinical operability and safety of the pre-dilution compounding strategy developed for the phentolamine-furosemide combination regimen.

Due to the extensive incompatibilities of furosemide injection with various clinical drugs, strict adherence to the drug package insert and authoritative compatibility databases (e.g., Trissel's Handbook on Injectable Drugs, Stabilis 4.0) is essential during practical application. Consulting pharmacists when necessary is also recommended. To minimize incompatibility risks, preventive measures such as administering drugs through independent pipelines, flushing the pipeline with appropriate infusion solvents before switching medications, utilizing precision infusion filter devices, and enhancing monitoring during clinical use should be implemented (31–33).

### 4.3 Limitations

This study has several limitations that should be acknowledged. First, all stability tests were conducted in static syringe systems under defined *in vitro* conditions (25 ± 1 °C, natural light, limited concentrations and two diluents), which cannot fully reproduce the dynamic and complex actual clinical infusion environment. Second, this study merely evaluated physicochemical stability of the admixtures, whereas microbiological stability, post- preparation sterility, infusion tubing compatibility, Y-site co-administration, drug adsorption to medical polymeric materials and *in vivo* safety assessments were not performed. Third, the white precipitate produced by direct mixing of undiluted injection was not chemically characterized by HPLC, LC-MS or other analytical techniques. It remains unclear whether the precipitate originates from precipitation of phentolamine mesylate or furosemide alone, or newly generated insoluble salts or complexes. Given the above constraints, the physico- chemical stability observed within 8 h only applies to the test conditions adopted in this experiment, and these data cannot be regarded as sufficient evidence to guarantee safe concurrent use in routine pediatric critical care.

## 5 Conclusions

In conclusion, phentolamine mesylate and furosemide injection with 0.9% NS or 5% GS can remain stable in the syringe at room temperature for 8 h. When preparing the diuretic mixture composed of phentolamine mesylate and furosemide for pediatric patients, the following points should be noted: (1) Strengthen aseptic operation during infusion preparation; (2) Use dilute mixing technique for infusion preparation. When preparing the infusion, the two drugs should be diluted with the infusion solution and mixed carefully. Alternatively, one drug can be added to a sufficient amount of infusion and mixed evenly before adding the other drug; (3) Perform routine visual inspection during infusion formulation and use, and the diuretic mixture should be administered immediately to maximize therapeutic efficacy and minimize the risk of adverse events in children.

## Data Availability

The original contributions presented in the study are included in the article/Supplementary Material, further inquiries can be directed to the corresponding author.
